# Philadelphia Chromosome-positive Acute Myeloid Leukemia With e1a3 *BCR-ABL1* Fusion Transcript

**DOI:** 10.1097/HS9.0000000000000484

**Published:** 2020-10-14

**Authors:** Julia W. Sheets, Patrick Eulitt, Rong He, Horatiu Olteanu, Catherine C. Coombs, Matthew C. Foster, Nathan D. Montgomery, Joshua F. Zeidner

**Affiliations:** 1 Lineberger Comprehensive Cancer Center, University of North Carolina, Chapel Hill, North Carolina, USA; 2 Department of Laboratory Medicine and Pathology, Mayo Clinic, Rochester, Minnesota, USA.; 3 Department of Pathology and Laboratory Medicine, University of North Carolina, Chapel Hill, North Carolina, USA

The Philadelphia (Ph) chromosome is a reciprocal translocation between chromosomes 9q34 and 22q11. The resultant BCR-ABL1 fusion protein can vary in size, primarily based on the specific breakpoint in the *BCR* gene, though less frequently also due to variation in the *ABL1* breakpoint. The 3 most common *BCR-ABL1* variants are identified by their protein molecular weight as p210 (e13a2 or e14a2), p190 (typically e1a2), and p230 (e19a2); alternative breakpoints are extremely rare.^1^ In myeloproliferative neoplasms, the presence of the Ph chromosome is diagnostic of chronic myeloid leukemia (CML); however, this translocation is also seen in approximately 2% to 3% of children and 20% to 30% of adults with acute lymphoblastic leukemia (ALL).^1^ In addition, the 2016 World Health Organization (WHO) Classification has recently added a provisional entity of Acute Myeloid Leukemia (AML) with *BCR-ABL1*, which is an extremely rare AML subtype.^2^ Tyrosine kinase inhibitors (TKIs) targeting the *BCR-ABL1* fusion gene are standard therapeutic agents effective for CML and used in combination with chemotherapy for patients with Ph chromosome positive (Ph+) ALL; however, there is little data regarding the use and efficacy of TKIs in Ph+ AML. We describe, to our knowledge, the first published case of a Ph+ AML patient with a *BCR-ABL1* breakpoint at e1a3 who subsequently developed an *ABL1* T315I mutation after treatment with dasatinib monotherapy.

A 68-year-old male with a history of dementia, coronary artery disease, type 2 diabetes mellitus and pulmonary alveolar proteinosis presented to the Emergency Department with a 2-week history of shortness of breath, malaise and anorexia. At presentation, a complete blood count revealed a marked leukocytosis (79.0 × 10^9^/L) with 68% blasts, absolute monocytosis (13.4 × 10^9^/L), mild eosinophilia (0.8 × 10^9^/L), and no basophils. The hemoglobin was 9.7 g/dL, and platelet count was 204 × 10^9^/L. Laboratory results from 5 months prior revealed normal blood counts. There was no splenomegaly on exam. A bone marrow (BM) biopsy revealed 78% blasts by manual aspirate differential in a hypercellular (95%) marrow. Flow cytometry of the peripheral blood revealed expression of CD11c, CD13, CD15, CD33, CD34, CD38, CD64, HLA-DR, and TdT with aberrant CD7, consistent with AML. Conventional cytogenetic analysis was positive for t(9;22)(q34;q11.2) as a sole abnormality in 18 of 22 metaphases. Three additional metaphases represented abnormal tetraploid subclones with 2 copies of the Ph chromosome. An interphase FISH assay with a Vysis BCR/ABL1 Dual Fusion translocation probe (Abbott, Abbott Park, IL) confirmed the presence of *BCR-ABL1* in 91% of the cells. A 35-gene next-generation sequencing panel revealed only a nonsense mutation in *stromal antigen 2* (*STAG2* R1242∗).

To better characterize the *BCR-ABL1* fusion detected by FISH, quantitative reverse transcriptase polymerase chain reactions (RT-PCR) studies targeting the canonical BCR-ABL1 p190 (e1a2) and p210 (e13a2 or e14a2) transcripts were performed on RNA extracted from the patient's BM sample. BCR-ABL1 transcripts were not detected in either the p190 (e1a2) or p210 RT-PCR assays. In order to identify an alternative breakpoint, a qualitative RT-PCR assay was performed, which can detect all known fusion BCR-ABL1 fusion transcripts. A variant BCR-ABL1 e1a3 transcript was detected, in which *BCR* exon 1 is spliced into *ABL1* exon 3 (Fig. 1).

**Figure 1:**
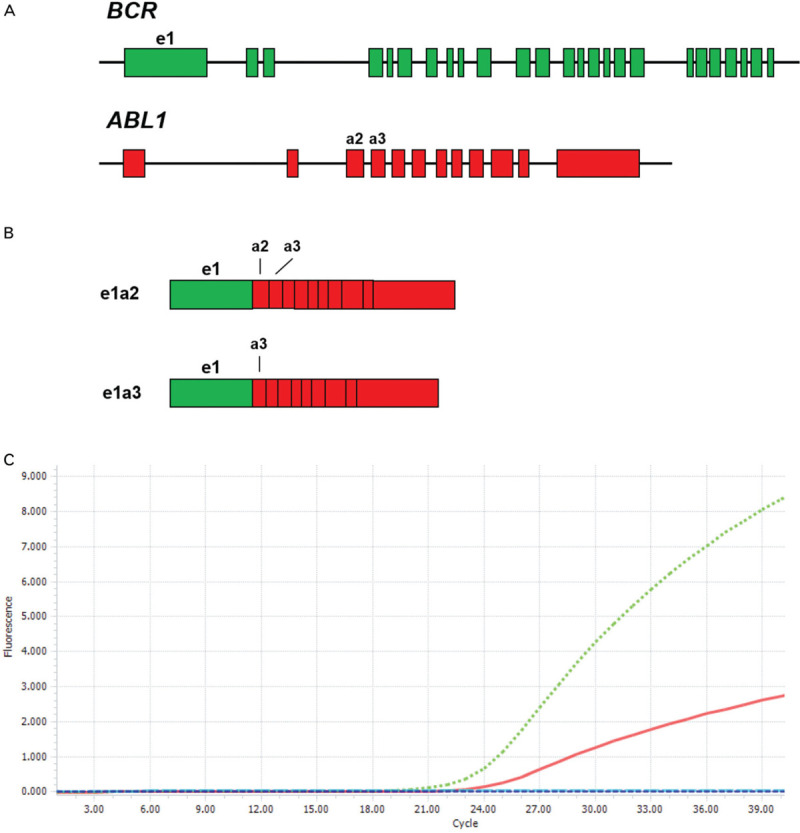
(A) Schematic representation of the exon/intron structures of *BCR* (top, exons green) and *ABL1* (bottom, exons red). *BCR* exon e1 and *ABL1* exons a2 and a3 are labeled. (B) Schematic representation of the e1a2 (top) and e1a3 (bottom) *BCR-ABL1* fusion transcripts. (C) RT-PCR plot of internal control *GUSB* (green line) and *BCR-ABL1* e1a3 (red line), confirming the presence of an e1a3 fusion.

Due to the patient's comorbidities and poor performance status, he was determined not to be a suitable candidate for induction chemotherapy. He was started on dasatinib 140 mg daily (off-label use) for Ph+ AML. The patient achieved a complete hematologic response (WBC 2.5 × 10^9^/L, ANC 1.1 × 10^9^/L, hemoglobin 9.8 g/dL and platelet count 306 × 10^9^/L) 57 days after initiating dasatinib therapy. After 3 months on dasatinib therapy, bone marrow biopsy revealed normocellular bone marrow (30%–40%) with trilineage hematopoiesis and 4% blasts indicating a CR. Interphase FISH for *BCR-ABL1* fusion remained low positive (3% of 200 cells scored) and flow cytometric minimal residual disease testing revealed 0.04% aberrant myeloblasts. The patient was deemed to be a very high-risk candidate for allogeneic stem cell transplant (alloSCT) due to his comorbidities and declined further work-up.

After 7 months on dasatinib, the patient presented with bilateral pleural effusions and acute kidney injury. Thoracentesis was performed, and 21% blasts were identified in the pleural fluid. Peripheral blood counts remained normal with only rare blasts. Bone marrow biopsy confirmed relapsed AML with 41% blasts by manual aspirate differential. *BCR-ABL1* fusion was detected in 52% of cells by interphase FISH. TKI resistance mutation testing was positive for an *ABL1* T315I mutation in the *BCR-ABL1* kinase domain. At diagnosis, the T315I mutation was not detected using a hybrid capture-based next-generation sequencing assay with a lower limit of detection of 0.5% VAF. Dasatinib was discontinued, and ponatinib 30 mg daily was initiated, which was increased to 45 mg daily after 1 month (off-label use for Ph+ AML). After 2 months on ponatinib, bone marrow biopsy revealed normocellular bone marrow with 1% blasts indicating CR. FISH results confirmed *BCR-ABL1* in 10% of interphase cells and MRD by flow cytometry was positive at 0.9%. Blood counts remained normal and repeat peripheral blood FISH results after 6 months of ponatinib therapy revealed *BCR-ABL1* in 6% of interphase cells revealing ongoing MRD. Unfortunately, this patient developed relapsed AML 8 months after initiating ponatinib. He subsequently received palliative care and died 17 months after initial diagnosis.

This is the first published case of Ph+ AML with the e1a3 *BCR-ABL1* variant transcript. While AML with *BCR-ABL1* is rare, it has been described in 0.5% to 3% of cases of AML and is considered adverse-risk by both the European Leukemia Net and National Cancer Center Network Guidelines.^3–5^ While it is clinically challenging to distinguish between CML in blast phase and de novo Ph+ AML, the patient meets WHO 2016 criteria for AML with *BCR-ABL1* with previously normal blood counts and no basophilia or splenomegaly at diagnosis, which has been associated with a diagnosis of AML with BCR-ABL1 rather than CML-blast crisis.^14^ The majority of Ph+ AML cases reported in the literature have been associated with the p190 transcript (e1a2), p210 transcript, or co-expression of both transcripts.^3^ The rare e6a2 transcript has also been described in 5 cases of Ph+ AML and has been associated with particularly aggressive disease.^6^

In this case, the e1a3 BCR-ABL variant was identified by RT-PCR as the sole *BCR-ABL1* transcript. The e1a3 transcript has been described in 7 cases of chronic phase CML (Table 1).^7–10^ In the 6 cases that were followed clinically, all 5 patients that received TKI therapy achieved either major molecular response or complete cytogenetic response.^7,8,10^ The e1a3 transcript has also been described in one case of CML that progressed to lymphoid blast crisis.^11^ In the ALL setting, the e1a3 transcript has been described in 20 cases of Ph+ ALL and has been associated with an aggressive clinical course with 44% of patients having relapsed or refractory disease (Table 1).^13^ To date, the e1a3 BCR-ABL variant has not been described in AML.

**Table 1:**
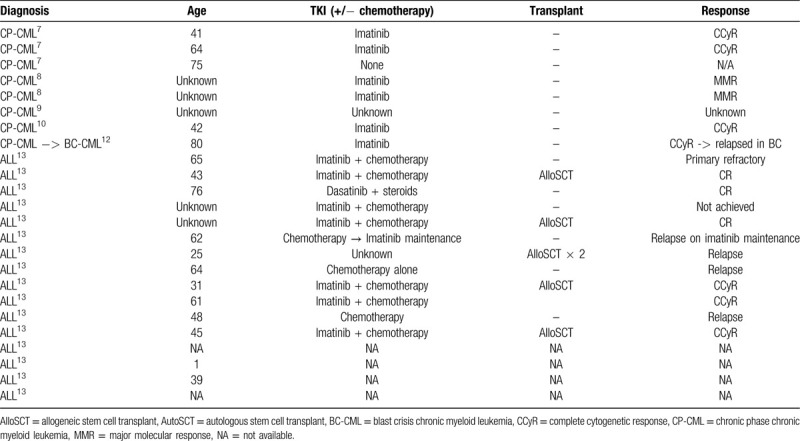
Cases of e1a3 Transcripts Reported in Literature.

The efficacy of TKI therapy in combination with chemotherapy for Ph+ ALL has been extensively investigated for over 10 years.^13^ Given the rare incidence of Ph+ AML, it is unknown whether TKIs have similar clinical efficacy in Ph+ AML. In a multi-institutional analysis of patients with Ph+ AML, 7 patients were treated with imatinib (5 in combination with chemotherapy, 2 with TKI alone).^14^ The median duration of response was only 2.5 months and median overall survival amongst all 16 Ph+ AML patients in that series was 9 months.^14^ Of note, these patients were all treated prior to the approval of second-generation TKIs. In a later review series of 21 patients with Ph+ AML treated with TKIs, most patients were treated with standard induction chemotherapy with the addition of a TKI and 38% of patients underwent allogeneic SCT^15^ (Supplementary Table). Of the patients unfit for transplant who received maintenance treatment with TKI alone, 92% achieved CR with median survival of 18.5 months.^15^ Only 2 patients in this series did not receive standard induction chemotherapy, and both were treated with TKI alone. One patient achieved a CR with single-agent TKI induction and consolidation while the other patient relapsed after allogeneic SCT and died 16 months after diagnosis.^15^ Resistance mutation data was not reported in these analyses.

The response to TKIs in the setting of rare transcripts in Ph+ AML has only been reported for the e6a2 transcript. In reported cases of AML with *BCR-ABL1* with the rare e6a2 transcript, 3 patients underwent allogeneic SCT and achieve sustained response with second-generation TKIs following transplant. The fourth patient had a particularly aggressive course and was refractory to TKIs.^6^ Therefore, while there appears to be a role for TKIs in AML with *BCR-ABL1*, further studies are needed to elucidate the optimal agent and dosing strategy, particularly in the setting of patients not suitable for intensive induction chemotherapy.

In summary, we report the first published case of a patient with AML with *BCR-ABL1* with the rare e1a3 variant. This patient received single agent dasatinib 140 mg daily and achieved MRD+ CR after 3 months of therapy. While this patient relapsed on dasatinib with a T315I mutation, he again achieved CR (with MRD) on ponatinib but subsequently relapsed and unfortunately died of progressive AML. Given the extremely rare incidence of the e1a3 Ph chromosome, it is unclear if TKI therapy is effective for this cohort of patients; nonetheless, limited data available suggests that TKIs may be effective alone or in combination with chemotherapy for AML with *BCR-ABL1* (Supplementary Table). Of note, we recommend interpreting this data with caution given the exceedingly positive results, which may be biased by the preferential publication of cases with favorable results. While AML with *BCR-ABL1* is a rare entity, given the therapeutic implications, we recommend performing FISH analysis for t(9;22) in patients in whom routine cytogenetics is not readily assessable or in patients with normal karyotypes with no known driver mutations. An international consortium would be best suited to derive further data and assess different therapeutic strategies in patients with AML with *BCR-ABL1*. All patients with AML with *BCR-ABL1* detected by FISH should have PCR performed to detect the specific *BCR-ABL1* transcript affected. If BCR-ABL1 PCR is negative for the most common BCR-ABL1 transcripts (ie, P190, P210, P230) then qualitative analysis for rare fusion transcripts should be performed. Patients with rare *BCR-ABL1* fusion variants should be managed similarly to patients with common transcripts, including with TKIs; however, further study is warranted to determine whether specific *BCR-ABL1* fusion transcripts may impact duration of TKI response and overall prognosis.

## Disclosures

JFZ has received honoraria from AbbVie, Agios, Celgene, Daiichi Sankyo, Genentech, Pfizer, and Takeda, has served as a consultant for AsystBio Laboratories, Celgene and Takeda, and has received research funding from AROG, Celgene, Forty Seven, Merck, Takeda, and Tolero Pharmaceuticals.

CCC has served as a consultant for Abbvie and Covance, has received honoraria from Abbvie, H3 Biomedicine, LOXO, Octapharma, and Pharmacyclics, and has received institutional research funding from AROG, Gilead, Incyte, LOXO, and H3 Biomedicine.

For the remaining authors, no relevant conflicts of interest were declared.

## Supplementary Material

Supplemental Digital Content
