# Supplementary material for: Philadelphia Chromosome-positive Acute Myeloid Leukemia With e1a3 BCR-ABL1 Fusion Transcript
Source: Hemasphere. 2020 Oct 14;4(6):e484. doi: 10.1097/HS9.0000000000000484 (PMC7566865; doi:10.1097/HS9.0000000000000484)
Supplement: Supplemental Digital Content [file hs9-4-e484-s001.docx]

Supplementary Table

| Patient Number | Chemotherapy | TKI Therapy | AlloSCT | Response | Survival |
| --- | --- | --- | --- | --- | --- |
| 1 | Yes | No | Yes | CR | Alive, 135 months |
| 2 | Yes | Imatinib 600 mg/day C | Yes | CR | Alive, 16 months |
| 3 | Yes | Imatinib 400 mg/day C, M | Yes | CR | Dead, 44 months |
| 4 | Yes | Imatinib 300 mg/day C, M | Yes | CR | Alive, 36 months |
| 5 | No | Imatinib 400-600 mg/day I, C, M | No | CR | Alive, 6 months |
| 6 | Yes | Imatinib C | No | Relapse | Dead, 10 months |
| 7 | No | Imatinib I, Dasatinib C | Yes | Relapse | Dead, 16 months |
| 8 | Yes | Imatinib I, C, M | No | CR | Alive, 71 months |
| 9 | Yes | Imatinib I, C, M | No | CR | Alive, 36 months |
| 10 | Yes | Imatinib I, C, M | No | CR | Alive, 32 months |
| 11 | Yes | Imatinib I, C, M | No | CR | Alive, 28 months |
| 12 | Yes | Imatinib 600 mg/day C, M | No | CR | Alive, 15 months |
| 13 | Yes | Imatinib 600 mg/day at relapse, C, M | No | CR2 | Alive, 48 months |
| 14 | Yes | Imatinib 400-600 mg/day C, M | No | CR | No data |
| 15 | Yes | Imatinib 300-600 mg/day I, C, M | No | CR | Alive, 18 months |
| 16 | Yes | Imatinib 400-600 mg/day C, M | No | CR | Alive, 19 months |
| 17 | Yes | Imatinib 400 mg/day C, M | No | CR | Alive, 12 months |
| 18 | Yes | Imatinib 600 mg/day C, M | No | CR | Alive, 10 months |
| 19 | Yes | Imatinib 400 mg/day I, C, relapse | Yes | CR2 | Alive, 18 months |
| 20 | Yes | Imatinib 400 mg/day C | Yes | CR | Alive, 25 months |
| 21 | Yes | Imatinib 400 mg/day C | Yes | CR | Alive, 9 months |
| 22 | Yes | Imatinib 400 mg/day C | Yes | CR | Alive, 6 months |
| 23 | Yes | Imatinib 400 mg/day C | Yes | CR | Alive, 6 months |

Treatment and Outcomes of Ph+ AML Cases Treated with TKI Therapy Reported in the Largest Case Series to Date [15]

Table Legend

I: Induction

C: Consolidation

M: Maintenance

CR: Complete Remission
